# Strength, Jumping, and Change of Direction Speed Asymmetries Are Not Associated With Athletic Performance in Elite Academy Soccer Players

**DOI:** 10.3389/fpsyg.2020.00175

**Published:** 2020-03-03

**Authors:** Javier Raya-González, Chris Bishop, Pedro Gómez-Piqueras, Santiago Veiga, David Viejo-Romero, Archit Navandar

**Affiliations:** ^1^Faculty of Health Sciences, Universidad Isabel I, Burgos, Spain; ^2^Faculty of Science and Technology, London Sport Institute, Middlesex University, London, United Kingdom; ^3^Faculty of Physical Education, Universidad de Castilla-La Mancha, Castilla-La Mancha, Spain; ^4^Health and Human Performance Department, Universidad Politécnica de Madrid, Madrid, Spain; ^5^Faculty of Sport Sciences, Universidad Europea de Madrid, Madrid, Spain

**Keywords:** fitness testing, interlimb differences, performance reduction, power, youth

## Abstract

The aims of the present study were 2-fold: (1) to measure interlimb asymmetries from a battery of fitness tests in youth soccer players and (2) to determine the association between asymmetry and measures of athletic performance. Sixteen elite youth soccer players (14.7 ± 0.2 years) performed a single-leg Abalakov test (ABK), change of direction (COD) test over 10 m (5 + 5) and 20 m (10 + 10), and an iso-inertial power test. Subjects also performed 10-, 20-, and 30-m sprints and a bilateral countermovement jump, which were correlated with all ABK, COD, and iso-inertial asymmetry scores. A one-way repeated-measures analysis of variance showed significant differences between interlimb asymmetry scores across multiple tests (*p* < 0.05), with the iso-inertial power test presenting the greatest magnitude of asymmetry, whereas individual data highlighted substantially greater interindividual differences in each test. Pearson *r* correlations showed no significant relationships (*p* > 0.05) between the different interlimb asymmetry scores, and between asymmetry scores and athletic performance. These findings show the test-specific nature of asymmetries in youth soccer players, with the iso-inertial power test being the most sensitive in detecting asymmetry. Moreover, the results obtained suggest that inherent asymmetry in young soccer players did not negatively impact their performance.

## Introduction

For team sport athletes, the majority of high-intensity actions occur unilaterally (e.g., sprinting, changing direction, kicking, and jumping) (Gonzalo-Skok et al., 2017; Bishop et al., 2018a). A recent study noted that these actions were unlikely to occur in an equal amount on each limb in professional soccer players (Bishop et al., 2019a); thus, the presence of interlimb asymmetry is to be somewhat expected. This is further supported in previous research from Hart et al. (2016), who showed that asymmetry is a by-product of playing sport, noting that positional differences are a contributing factor to the prevalence of asymmetry as well. However, the prevalence of asymmetry alone does little to inform practitioners about whether targeted training interventions are required from an injury reduction or performance enhancement perspective.

Recently, there has been a rise in studies investigating the association between interlimb asymmetry and measures of athletic performance. For example, Bishop et al. (2018b) showed that jump height asymmetries from the unilateral countermovement jump (CMJ) were associated with slower 5-m (*r* = 0.49), 10-m (*r* = 0.52), and 20-m (*r* = 0.59) sprint times in elite youth female soccer players. Additionally, in elite academy soccer players of multiple age groups, ranging from younger than 16 to younger than 23 years old, Bishop et al. (2019b) showed that jump height asymmetry (again from the unilateral CMJ) was associated with slower times in 5- (*r* = 0.60–0.86), 10- (*r* = 0.54–0.87), 20-m (*r* = 0.56–0.79) sprints, and it also affected performance in the 5-0-5 test in either limb (*r* = 0.61–0.85). In contrast, Lockie et al. (2014) reported jump height and distance asymmetries during the unilateral CMJ (10.4%), lateral jump (5.1%), and broad jump (3.3%) tests in male collegiate athletes. No significant relationships were reported with linear speed or change of direction (COD) speed tests. Similarly, Dos’Santos et al. (2017) reported distance asymmetries of 5 to 6% for the single and triple hop for distance tests in male collegiate athletes and found no significant relationships between the two COD speed tests. Hence, in lieu of the available evidence, results demonstrate inconclusive findings when aiming to determine the association between interlimb asymmetry and measures of athletic performance. Thus, further research in this area is warranted.

In addition to this conflicting evidence, there is a paucity of studies investigating the relationship between strength/power asymmetry and performance tests. Previous research supports the notion that strength asymmetry is negatively correlated with jump performance (Bailey et al., 2013), speed and COD speed (Coratella et al., 2018), and kicking accuracy (Hart et al., 2014). However, these studies analyzed the strength asymmetry scores through the concentric phase of the movement, or even with isometric contractions, despite the fact that most of the sportive actions occur by combining concentric and eccentric contractions (i.e., the stretch-shortening cycle) (Nuñez and Sáez de Villarreal, 2017). An interesting alternative for the assessment of asymmetry would be to use flywheel devices, because these allow to apply load during both phases of the movement (i.e., concentric and eccentric) (Beato et al., 2019). In addition, to the authors’ knowledge, only one previous study has analyzed whether asymmetries observed in an iso-inertial power test influence the athletic performance (Madruga-Parera et al., 2019), showing no significant relationships between them. However, the authors used specific tests (i.e., shuffle lateral and crossover steps) in youth tennis players, and no research has studied the relationship between the level of the asymmetry obtained in an iso-inertial soccer-related test (i.e., lateral squat) and the performance in elite young soccer players.

Therefore, the aims of the present study were 2-fold: (1) to measure interlimb asymmetries from a battery of fitness tests in youth soccer players and (2) to determine the association between asymmetry and measures of athletic performance. Owing to the conflicting evidence, developing a logical hypothesis was challenging, however, it was thought that larger asymmetries would be associated with reduced athletic performance.

## Materials and Methods

### Participants

Sixteen elite U15 male soccer players (age = 14.7 ± 0.2 years, height = 169.1 ± 8.3 cm, body mass = 56.6 ± 9.7 kg, body mass index = 20.1 ± 1.8 kg/m^2^) volunteered to participate in the study (*post hoc* statistical power >0.80). The players played in the highest division corresponding to their age-category level in Spain. Prior to participating in the study, each player completed a questionnaire about their medical and injury history. Goalkeepers were excluded from the study sample, and players who did not complete all tests were omitted in the subsequent statistical analysis. The inclusion criteria were to complete all the tests and to not have been injured during the last month before the investigation. Prior to initiating the study, participants were fully informed about the protocol to follow, and their assent was collected. Additionally, their parents/legal guardians filled out informed consents as they were younger than 18 years. All participants were free to leave the study at any time without any penalty. The study followed the guidelines set out in the Declaration of Helsinki (2013) and was approved by the university’s research ethics.

### Procedures

All data collection sessions were scheduled before the regular soccer sessions with testing taking place over 2 weeks. A familiarization period took place during the first weeks (two sessions), in order to avoid any learning effects (Castillo et al., 2019), while during the second week, the participants performed the two data collection sessions with 48 h of separation between sessions. Participants were required to complete the jump and power test during the first data collection session, and the linear and COD sprint test in the second one. This order was agreed with the clubs as it was deemed to minimize the accumulation of fatigue and therefore most likely to maximize performances across all tests. During the experimental period, the concerned players were instructed to have their last meal 3 h before the beginning of the tests, not to drink any caffeinated beverages or to perform intense physical exercise. All tests were performed at the same time, in the field of artificial grass where the team had their training session, with the training gear and footwear normally used by the player in the training. In addition, all these sessions were supervised by the accredited strength and conditioning coaches, who verbally discussed with each participant to ensure both parties were satisfied with requirements before data collection. Before each data collection session, a standardized warm-up was performed, consisting of 3 min of continuous, low-intensity running; joint mobility exercises; and jump and sprint actions over distances of 10 to 30 m.

### Vertical Jump Tests

After the standardized warm-up, the players performed three bilateral CMJ and three unilateral Abalakov jumps (ABK) with each limb, separated by 45 s of passive recovery (Núñez et al., 2018). During the CMJ, all participants were instructed to place their hands on their hips, which was followed by a vertical jump at maximal effort and landing in a vertical position, with their knees being flexed after landing (Sáez de Villarreal et al., 2015). However, during ABK, the swinging of the arms was allowed. All the jumps were performed on a platform with infrared rays (Optojump Next; Microgate^®^, Bolzano, Italy), and the jump height (cm) was recorded. The best of the performances of each test was selected for the subsequent statistical analysis.

### Iso-Inertial Power Test

**FIGURE 1:**
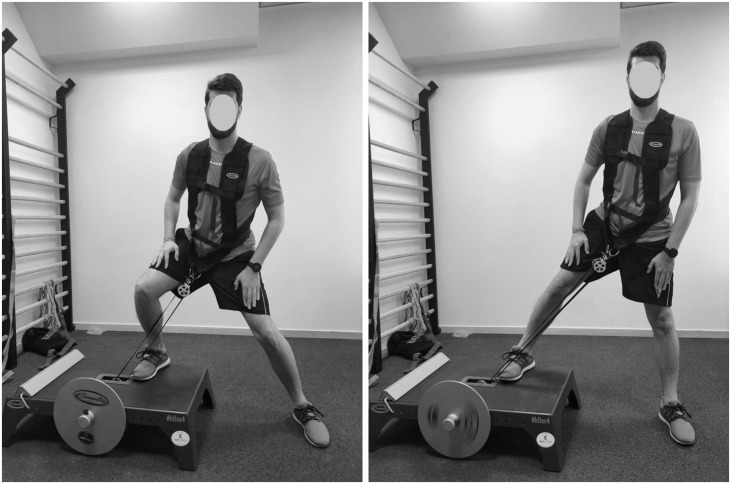
The iso-inertial power test.

Following the jump test, the power test was conducted using a flywheel device (K-Box 3; Exxentric^®^, Stockholm, Sweden). All players performed 2 sets of 6 repetitions of the lateral squat exercise (Figure 1) with each leg (inertia 0.10 kg/m^2^), allowing a rest of 4 min between each attempt (Sabido et al., 2017). Mean and peak power were measured by means of a rotary encoder (SmartCoach^TM^ Power Encoder; SmartCoach Europe AB, Stockholm, Sweden) using its associated software (v5.6.0.8) in both concentric and eccentric phases, being the sum of both values calculated. The inertial load used for this assessment was chosen because previous studies have shown that power values are higher with lower loads (Sabido et al., 2017). To avoid possible fatigue effects, 50% of the participants started with their right limb, and the other 50% started with their left limb. The best result obtained in each test was selected for the subsequent statistical analysis.

### Linear Sprint Test

Participants were assessed over 30 m with split times on 10 and 20 m. Four pairs of photoelectric cells (Polifemo Light Radio; Microgate^®^) were used to record the sprint times. The starting position was placed 0.5 m before the first timing gate, and players started when ready eliminating reaction time. Two trials with a rest of 2 min between each sprint were completed, and the fastest time was considered for the subsequent statistical analysis.

### Change of Direction Sprint Test

**FIGURE 2:**
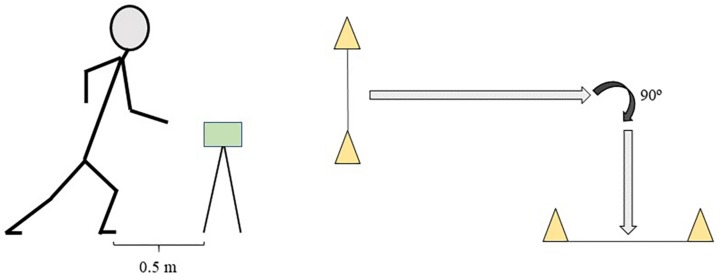
Schematic representation of change of direction tests configuration.

After the linear sprint test, participants performed a total of four sprints with a COD involved. There were two sprints of 10 m (5 + 5 m) and two sprints of 20 m (10 + 10 m) with a COD of 90° (Figure 2). Each set of sprints was repeated so that the player changed direction to the right twice and changed direction to the left twice (Hader et al., 2015), and 50% of the participants started with their right limb, and the other 50% started with their left limb. A recovery time of 2 min was allowed between each sprint. At the beginning of each sprint, the front foot was placed 0.5 m before the first photocell (Polifemo Light Radio; Microgate^®^). For the subsequent statistical analysis, the fastest time of each test was chosen.

### Statistical Analysis

Results are presented as mean ± standard deviations. All the dependent variables obtained from the tests were tested for normality using the Shapiro-Wilk test, and all the variables obtained were normally distributed. Within-session reliability of test measures was computed using the intraclass correlation coefficient (ICC) with absolute agreement and the coefficient of variation (CV). Interpretation of ICC and the 95% confidence interval were calculated and categorized as excellent (0.90–1.00), good (0.75–0.9), moderate (0.50–0.75), or poor (<0.50) (Koo and Li, 2016), considering CV values lower than 10% as acceptable (Cormack et al., 2008). In each unilateral test, the limb where a higher score was obtained was determined as the stronger limb, and the other limb was denoted as the weaker limb, and subsequently, interlimb asymmetries were calculated using a standard percentage difference equation for all the tests: (score in stronger limb − score in weaker limb)/(score in stronger limb) × 100 (Impellizzeri et al., 2007). When depicting interlimb differences individually, the use of an “IF function” in Microsoft Excel was added to the end of the formula: ^∗^IF (left < right, 1,−1) (Bishop et al., 2018a), in order to show the direction of asymmetry (i.e., which leg produced the larger score) without altering the magnitude. A one-way repeated-measures analysis of variance was conducted to determine the systematic bias between mean asymmetry scores, with statistical significance set at *p* < 0.05. Pearson *r* correlations were conducted to establish the relationship between interlimb asymmetries and fitness test scores, with statistical significance set at *p* < 0.05. The following scale of magnitudes was used to interpret the correlation coefficients: <0.1, trivial; 0.1–0.3, small; 0.3–0.5, moderate; 0.5–0.7, large; 0.7–0.9, very large; and >0.9, nearly perfect (Hopkins et al., 2009). All statistical tests were performed using the software package SPSS version 24.0 (SPSS Inc., Chicago, IL, United States).

## Results

Within-session reliability data are presented in Table 1 and show that all data reported an excellent reliability except for 10-m linear speed (ICC = 0.75), whereas acceptable CV for all the tests (<10%) was obtained. In addition, significantly higher values (*p* < 0.05) were observed in ABK, mean power, and peak power interasymmetry scores in comparison to COD5 and COD10, without differences between the other tests.

**TABLE 1:** Mean test scores ± standard deviations (SDs), test reliability (95% CIs), and mean interlimb asymmetry values.

Fitness test	Mean ± SD	ICC (95% CI)	CV (95% CI)	Mean asymmetry (%)
ABK-S (cm)	24.543.61	0.96 (0.93–0.98)	2.8 (2.1–4.3)	11.955.82*
ABK-W (cm)	21.552.86	0.96 (0.92–0.98)	3.2 (2.4–4.9)	
COD5-S (s)	2.580.12	0.94 (0.86–0.97)	1.2 (0.9–1.8)	4.602.51
COD5-W (s)	2.460.12	0.95 (0.89–0.98)	1.3 (1.0–2.0)	
COD10-S (s)	4.040.14	0.96 (0.91–0.98)	0.9 (0.7–1.4)	3.021.74
COD10-W (s)	4.170.14	0.93 (0.85–0.97)	0.8 (0.6–1.3)	
**Lateral squat:**				
Mean power-S (W)	301.70114.86	0.98 (0.95–0.99)	3.9 (2.9–6.0)	21.2715.55*
Mean power-W (W)	241.60114.19	0.99 (0.97–1.00)	2.3 (1.8–3.5)	21.6818.85*
Peak power-S (W)	524.83198.13	0.97 (0.92–0.99)	7.3 (5.4–11.2)	
Peak power-W (W)	416.41202.41	0.99 (0.98–1.00)	2.9 (2.2–4.5)	
**Speed:**				
10 m (s)	1.760.07	0.75 (0.48–0.88)	2.3 (1.7–3.5)	—
20 m (s)	3.090.11	0.93 (0.85–0.97)	1.1 (0.8–1.6)	—
30 m (s)	4.310.17	0.97 (0.93–0.99)	0.7 (0.5–1.0)	—
Countermovement jump (cm)	37.234.98	0.96 (0.92–0.98)	3.4 (2.5–5.2)	—

* Higher interlimb asymmetry scores in comparison to COD tests (*p* < 0.05). ICC, intraclass correlation coefficient; CV, coefficient of variation; CI, confidence interval; ABK, Abalakov test; S, strong; W, weak; COD, change of direction.

**FIGURE 3:**
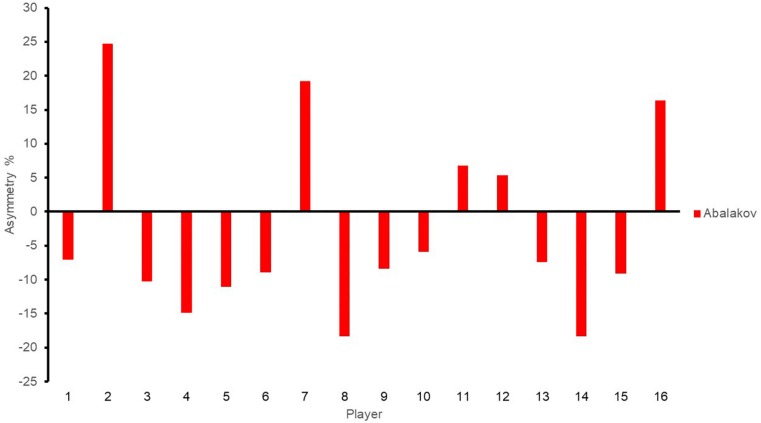
Individual asymmetry data for Abalakov test. Above the line indicates raw score is greater on the right limb, and below the line indicates raw score is greater on the left limb. ABK, Abalakov test.

**FIGURE 4:**
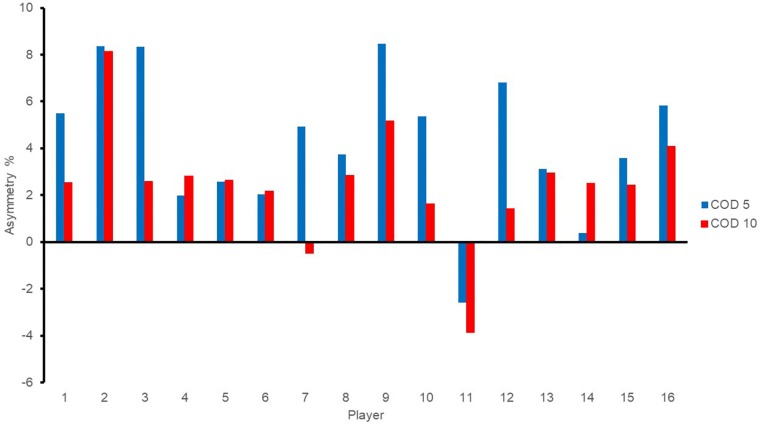
Individual asymmetry data for change of direction tests. Above the line indicates raw score is greater on the right limb, and below the line indicates raw score is greater on the left limb. COD, change of direction.

**FIGURE 5:**
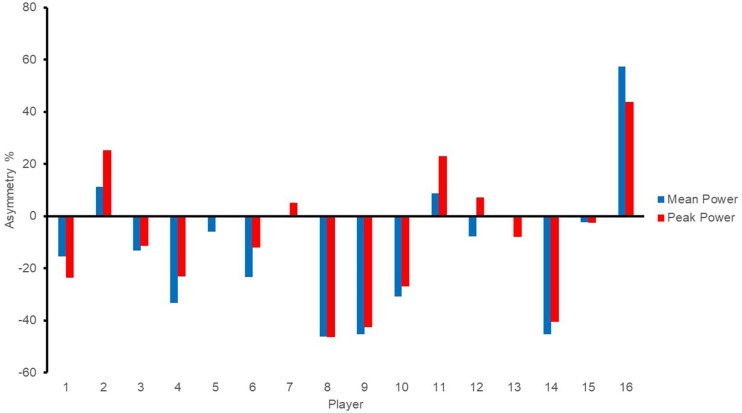
Individual asymmetry data for iso-inertial power test. Above the line indicates raw score is greater on the right limb, and below the line indicates raw score is greater on the left limb.

Individual asymmetry values for each player are presented in Figures 3–5 for jump height (ABK), COD (i.e., 5 and 10), and power (i.e., mean and peak). Individual asymmetry values ranged from 5.86 to 24.65% in ABK, 0.37 to 8.46% in COD5, 0.48 to 8.13% in COD10, 0.30 to 46.40% in mean power, and 0.14 to 57.37% in peak power.

Pearson *r* correlations between interlimb asymmetry scores across tests are shown in Table 2; no significant relationships were present between tests. Pearson *r* correlations between interlimb asymmetry scores and fitness tests are shown in Table 3. No significant relationships were found between interlimb asymmetry scores and either speed or jump performance.

**TABLE 2:** Pearson *r* correlations (95% confidence intervals) between the different interlimb asymmetry scores.

Tests	ABK	COD5	COD10	MP
COD5	0.01 (±0.43)	—		
COD10	0.40 (±0.37)	0.40 (±0.37)	—	
MP	0.34 (±0.39)	0.09 (±0.42)	0.40 (±0.37)	—
PP	0.26 (±0.40)	−0.03 (±0.43)	0.19 (±0.41)	0.90 (±0.09)*

* Statistical significance at *p* < 0.01. ABK, Abalakov test; COD, change of direction; MP, mean power; PP, peak power.

**TABLE 3:** Pearson *r* correlations (95% confidence intervals) between the different interlimb asymmetry scores and speed and jump tests.

Asymmetry	S10	S20	S30	CMJ
ABK	−0.14 (±0.42)	−0.15 (±0.42)	−0.19 (±0.41)	0.17 (±0.42)
COD5	−0.43 (±0.36)	−0.38 (±0.38)	−0.32 (±0.39)	0.42 (±0.36)
COD10	−0.19 (±0.41)	−0.24 (±0.41)	−0.24 (±0.41)	0.04 (±0.43)
MP	0.17 (±0.42)	0.22 (±0.41)	0.16 (±0.42)	0.26 (±0.40)
PP	0.29 (±0.40)	0.31 (±0.39)	0.22 (±0.41)	−0.15 (±0.42)

*ABK, Abalakov test; COD, change of direction; MP, mean power; PP, peak power.*

## Discussion

The aims of the present study were to measure interlimb asymmetries from a battery of fitness tests in youth soccer players and to determine the association between asymmetry and measures of athletic performance. Results showed varying magnitudes of asymmetry across tests, the largest of which was during the iso-inertial power tests. Abalakov test and iso-inertial power tests showed larger asymmetry scores when comparing to the COD tests, and the correlations between asymmetry indexes were not significant. In addition, no significant relationship between asymmetry scores and athletic performance was observed.

All tests showed excellent reliability, except for 10 m, which showed an acceptable variability (<10%). These findings suggest the data being presented in this article can be interpreted with confidence for further analysis (Turner et al., 2015). Previous research supports that the level of experience and the structured strength and conditioning training (inclusive of speed and jump training) performed during the season seem to contribute to the good reliability of the data (Bishop et al., 2019a). Regarding the asymmetry scores observed in the present study, iso-inertial power tests showed the largest asymmetries in both mean and peak power (21.27% ± 15.55% and 21.68% ± 18.85%, respectively) across the different proposed tests, whereas the COD test showed a lower magnitude of asymmetry (COD5: 4.60% ± 2.51% and COD10: 3.02% ± 1.74%) in comparison to the jump and iso-inertial power tests, which is in agreement with previous research (Fort-Vanmeerhaeghe et al., 2015; Dos’Santos et al., 2018; Madruga-Parera et al., 2019). The COD tests’ inability to detect asymmetries could be due to two reasons: first, there is a strong linear speed component during COD, mainly in COD10 (Madruga-Parera et al., 2019), and second, sprint times are far more replicable than power outputs during jump (Bishop et al., 2019a) or iso-inertial tests.

No significant relationships were present between asymmetry scores, highlighting the independent nature of the selected tests in elite youth male soccer players (Table 2). This finding is supported by previous studies (Loturco et al., 2018; Bishop et al., 2019c), which stated a lack of relationships between different asymmetry scores in both female and male elite senior soccer players. Furthermore, in a recent study, Bishop et al. (2018a) observed that when comparing asymmetry scores across multiple tests levels of agreement were typically poor. This information suggests that asymmetries are independent of each other, reflecting the necessity to introduce different tests to provide a holistic picture of the asymmetries in the soccer players, as well as to preclude the use of a single test to screen for interlimb asymmetry. Given similar findings have been shown across multiple populations (Bishop et al., 2018a, 2019a; Loturco et al., 2018), it seems prudent to suggest that the lack of association between asymmetry in different tasks has less to do with youth male soccer players, but more to do with the variable nature of asymmetry itself.

As the interlimb asymmetry scores vary according to the test used, not all players respond equally to the same test in terms of asymmetry. Regarding this, some authors (Bishop et al., 2018a) have postulated that individual asymmetry analysis is key, owing to the high variability for the mean asymmetry scores (Table 1). In this sense, the largest mean asymmetry value for the analyzed test ranged from 3.02 to 21.68%, however, it is clear from Figures 3–5 that many individual asymmetry values greatly surpassed this. In addition, individual analysis provides information about which asymmetry values favor the left limb (as represented by negative scores) and which favor the right limb (positive asymmetry outcome) for each player, highlighting how a limb may be favored over the other from task to task (Bishop et al., 2018a). This individual information seems to be critical to perform specific training interventions, in order to reduce optimally interlimb asymmetries, because thresholds of greater than 10% are to be accepted as cutoffs where reduced performance (Bishop et al., 2018c) and increased risk of injury are present (Rohman et al., 2015). Therefore, to deepen in the interlimb asymmetry knowledge, the athlete profile should present the individual data as well as mean values.

It is well documented that in this sport several high-intensity actions occur unilaterally, not being equal the implication of both limbs (Sabido et al., 2017), which lead to the presence of asymmetries in soccer players (Bishop et al., 2019a). Attending to this, previous studies performed with soccer players have reported that jump asymmetry impacts negatively in athletic performance (Bishop et al., 2019a, b). Conversely, in the present study, no significant relationships were observed between interlimb asymmetries from ABK and either speed or jump performance. These differences could be due to the different jump test used in previous studies (i.e., drop jump), which is characterized by the presence of a braking action with an immediate requirement to transition into high propulsive forces straight, something that does seem to affect COD performance (Jones et al., 2009). According to this, no significant correlations were presented between COD asymmetry and athletic performance. Although we hypothesized that the asymmetry scores obtained in the iso-inertial power test could influence athletic performance, our results revealed a lack of significant relationships. In this sense, only one previous study has used an iso-inertial device to assess interlimb asymmetries and its relationship with jump performance (Madruga-Parera et al., 2019), obtaining similar results. Given that asymmetries have no detrimental impact in young soccer players’ performance, even using iso-inertial devices for the assessment, it seems pertinent to assume that reducing asymmetry scores is not a relevant strategy to improve the performance in this specific population. Further studies should analyze the relationship between asymmetry and external match load (i.e., distance covered, high-speed running, etc.) in order to understand the relevance of asymmetry on match performance in young soccer players.

This study is not without limitations. The main one is that maturation stages have not been considered in this study; thus, an assumption was made that players were no longer circa peak height velocity, which may have influenced the results obtained in this study. Additionally, a small sample size of 16 subjects was used, so the results obtained should be taken with caution. Therefore, future research should aim to use a larger cohort of soccer players, as well as to include the maturation status of the participants.

## Conclusion

The findings from this study highlight that asymmetries vary across commonly used tests without significant relationships between each other, suggesting the necessity to apply a fitness test battery for youth soccer players in order to provide a holistic picture of the asymmetries in the soccer players. On the other hand, individual asymmetry scores were vastly different from mean values for all metrics, so practitioners should always consider the individual nature of asymmetries to perform specific training interventions on a more individual level. In addition, iso-inertial power test appears to be a highly sensitive test to detect asymmetries. However, this power test had no detrimental association with sprint and jumping abilities. These findings suggest that the reduction of interlimb asymmetries should not be expected to impact player performance in youth soccer players.

## Data Availability Statement

All datasets generated for this study are included in the article/supplementary material.

## Ethics Statement

The studies involving human participants were reviewed and approved by Universidad Isabel I. Written informed consent to participate in this study was provided by the participants’ legal guardian/next of kin.

## Author Contributions

JR-G conceived the research idea, collected the sample data, analyzed the data, and statistically interpreted the findings. JR-G, CB, SV, PG-P, and AN prepared the manuscript. All authors critically revised the manuscript, read, and approved the final version.

## Conflict of Interest

The authors declare that the research was conducted in the absence of any commercial or financial relationships that could be construed as a potential conflict of interest.

## References

[B1] Bailey C. Sato K. Alexander R. Chiang C.-Y. Stone M. H. (2013). Isometric force production symmetry and jumping performance in collegiate athletes. *J. Trainol.* 2 1–5. 10.17338/trainology.2.1_1

[B2] Beato M. McErlain-Naylor S. A. Halperin I. Dello Iacono A. (2019). Current evidence and practical applications of flywheel eccentric overload exercises as postactivation potentiation protocols: a brief review. *Int. J. Sports Physiol. Perform.* [Epub ahead of print]. 31743092 10.1123/ijspp.2019-0476

[B3] Bishop C. Berney J. Lake J. Loturco I. Blagrove R. Turner A. (2019a). Bilateral deficit during jumping tasks. *J. Strength Cond. Res.* [Epub ahead of print]. 10.1519/JSC.0000000000003075 30741876

[B4] Bishop C. Brashill C. Abbott W. Read P. Lake J. Turner A. (2019b). Jumping asymmetries are associated with speed, change of direction speed, and jump performance in elite academy soccer players. *J. Strength Cond. Res.* [Epub ahead of print]. 30707141 10.1519/JSC.0000000000003058

[B5] Bishop C. Lake J. Loturco I. Papadopoulos K. Turner A. Read P. (2018a). Interlimb asymmetries: the need for an individual approach to data analysis. *J. Strength Cond. Res.* [Epub ahead of print]. 10.1519/JSC.0000000000002729 33587548

[B6] Bishop C. Read P. McCubbine J. Turner A. (2018b). Vertical and horizontal asymmetries are related to slower sprinting and jump performance in elite youth female soccer players. *J. Strength Cond. Res.* [Epub ahead of print]. 10.1519/JSC.0000000000002544 29489719

[B7] Bishop C Turner A Maloney S Lake J Loturco I Bromley T (2019c). Drop jump asymmetry is associated with reduced sprint and change-of-direction speed performance in adult female soccer players. *Sports* 7:E29. 10.3390/sports7010029 30669686 PMC6359266

[B8] Bishop C. Turner A. Read P. (2018c). Effects of inter-limb asymmetries on physical and sports performance: a systematic review. *J. Sports Sci.* 36 1135–1144. 10.1080/02640414.2017.1361894 28767317

[B9] Castillo D. Domínguez R. Rodríguez-Fernández A. Raya-González J. (2019). Effects of caffeine supplementation on power performance in a flywheel device: a randomised, double-blind cross-over study. *Nutrients* 11:255. 10.3390/nu11020255 30678333 PMC6412282

[B10] Coratella G. Beato M. Schena F. (2018). Correlation between quadriceps and hamstrings inter-limb strength asymmetry with change of direction and sprint in U21 elite soccer-players. *Hum. Mov. Sci.* 59 81–87. 10.1016/j.humov.2018.03.016 29625360

[B11] Cormack S. J. Newton R. U. McGuigan M. R. Doyle T. L. A. (2008). Reliability of measures obtained during single and repeated countermovement jumps. *Int. J. Sports Physiol. Perform.* 3 131–144. 10.1123/ijspp.3.2.131 19208922

[B12] Dos’Santos T. Thomas C. Jones P. A. Comfort P. (2018). Assessing asymmetries in change of direction speed performance; application of change of direction deficit. *J. Strength Cond. Res.* 33 2953–2961. 10.1519/JSC.0000000000002438 29373434

[B13] Dos’Santos T. Thomas C. A. Jones P. Comfort P. (2017). Asymmetries in single and triple hop are not detrimental to change of direction speed. *J. Trainology* 6 35–41. 10.17338/trainology.6.2-35

[B14] Fort-Vanmeerhaeghe A. Montalvo A. M. Sitjà-Rabert M. Kiefer A. W. Myer G. D. (2015). Neuromuscular asymmetries in the lower limbs of elite female youth basketball players and the application of the skillful limb model of comparison. *Phys. Ther. Sport.* 16 317–323. 10.1016/j.ptsp.2015.01.003 26093377

[B15] Gonzalo-Skok O. Tous-Fajardo J. Suarez-Arrones L. Arjol-Serrano J. L. Casajús J. A. Mendez-Villanueva A. (2017). Single-leg power output and between-limbs imbalances in team-sport players: unilateral versus bilateral combined resistance training. *Int. J. Sports Physiol. Perform.* 12 106–114. 10.1123/ijspp.2015-0743 27140680

[B16] Hader K. Palazzi D. Buchheit M. (2015). Change of direction speed in soccer: how much braking is enough? *Kinesiology* 47 67–74.

[B17] Hart N. H. Nimphius S. Spiteri T. Newton R. U. (2014). Leg strength and lean mass symmetry influences kicking performance in Australian football. *J. Sports Sci. Med.* 13 157–165. 24570620 PMC3918553

[B18] Hart N. H. Nimphius S. Weber J. Spiteri T. Rantalainen T. Dobbin M. (2016). Musculoskeletal asymmetry in football athletes. *Med. Sci. Sport. Exerc.* 48 1379–1387. 10.1249/MSS.0000000000000897 26871989

[B19] Hopkins W. G. Marshall S. W. Batterham A. M. Hanin J. (2009). Progressive statistics for studies in sports medicine and exercise science. *Med. Sci. Sport. Exerc.* 41 3–13. 10.1249/MSS.0b013e31818cb278 19092709

[B20] Impellizzeri M. Rampinini E. Maffiuletti N. Marcora S. (2007). A vertical jump force test for assessing bilateral strength asymmetry in athletes. *Med. Sci. Sport. Exerc.* 39 2044–2050. 10.1249/mss.0b013e31814fb55c 17986914

[B21] Jones P. Bampouras T. M. Marrin K. (2009). An investigation into the physical determinants of change of direction speed. *J. Sports Med. Phys. Fit.* 49 97–104. 19188902

[B22] Koo T. K. Li M. Y. (2016). A guideline of selecting and reporting intraclass correlation coefficients for reliability research. *J. Chiropr. Med.* 15 155–163. 10.1016/j.jcm.2016.02.012 27330520 PMC4913118

[B23] Lockie R. G. Callaghan S. J. Berry S. P. Cooke E. R. A. Jordan C. A. Luczo T. M. (2014). Relationship between unilateral jumping ability and asymmetry on multidirectional speed in team-sport athletes. *J. Strength Cond. Res.* 28 3557–3566. 10.1519/JSC.0000000000000588 24942166

[B24] Loturco I. Pereira L. A. Kobal R. Abad C. C. C. Komatsu W. Cunha R. (2018). Functional screening tests: interrelationships and ability to predict vertical jump performance. *Int. J. Sports Med.* 39 189–197. 10.1055/s-0043-122738 29284166

[B25] Madruga-Parera M. Bishop C. Fort-Vanmeerhaeghe A. Beltran-Valls M. R. Skok O. G. Romero-Rodríguez D. (2019). Interlimb asymmetries in youth tennis players. *J. Strength Cond. Res.* [Epub ahead of print]. 31009431 10.1519/JSC.0000000000003152

[B26] Nuñez F. J. Sáez de Villarreal E. (2017). Does flywheel paradigm training improve muscle volume and force? A meta-analysis. *J. Strength Condition. Res.* 31 3177–3186. 10.1519/JSC.0000000000002095 29068866

[B27] Núñez F. J. Santalla A. Carrasquila I. Asian J. A. Reina J. I. Suarez-Arrones L. J. (2018). The effects of unilateral and bilateral eccentric overload training on hypertrophy, muscle power and COD performance, and its determinants, in team sport players. *PLoS One* 13:e0193841. 10.1371/journal.pone.0193841 29590139 PMC5874004

[B28] Rohman E. Steubs J. T. Tompkins M. (2015). Changes in involved and uninvolved limb function during rehabilitation after anterior cruciate ligament reconstruction: implications for Limb Symmetry Index measures. *Am. J. Sports Med.* 43 1391–1398. 10.1177/0363546515576127 25828078

[B29] Sabido R. Hernández-Davó J. L. Botella J. Navarro A. Tous-Fajardo J. (2017). Effects of adding a weekly eccentric-overload training session on strength and athletic performance in team-handball players. *Eur. J. Sport Sci.* 17 530–538. 10.1080/17461391.2017.1282046 28152673

[B30] Sáez de Villarreal E. Suarez-Arrones L. Requena B. Haff G. G. Ferrete C. (2015). Effects of plyometric and sprint training on physical and technical skill performance in adolescent soccer players. *J. Strength Cond. Res.* 29 1894–1903. 10.1519/JSC.0000000000000838 25635606

[B31] Turner A. Brazier J. Bishop C. Chavda S. Cree J. Read P. (2015). Data analysis for strength and conditioning coaches. *Strength Cond. J.* 37 76–83. 10.1519/SSC.0000000000000113

