# Corrigendum: Strength, Jumping, and Change of Direction Speed Asymmetries Are Not Associated With Athletic Performance in Elite Academy Soccer Players

**DOI:** 10.3389/fpsyg.2020.00518

**Published:** 2020-03-20

**Authors:** Javier Raya-González, Chris Bishop, Pedro Gómez-Piqueras, Santiago Veiga, David Viejo-Romero, Archit Navandar

**Affiliations:** ^1^Faculty of Health Sciences, Universidad Isabel I, Burgos, Spain; ^2^Faculty of Science and Technology, London Sport Institute, Middlesex University, London, United Kingdom; ^3^Faculty of Physical Education, Universidad de Castilla-La Mancha, Castilla-La Mancha, Spain; ^4^Health and Human Performance Department, Universidad Politécnica de Madrid, Madrid, Spain; ^5^Faculty of Sport Sciences, Universidad Europea de Madrid, Madrid, Spain

**Keywords:** fitness testing, interlimb differences, performance reduction, power, youth

In the original article, the specific contributions of the authors Santiago Veiga and Pedro Gómez-Piqueras were not included in the Author Contributions statement. The corrected Author Contributions Statement appears below.

## Author Contributions

JR-G conceived the research idea, collected the sample data, analyzed the data, and statistically interpreted the findings. JR-G, CB, SV, PG-P, and AN prepared the manuscript. All authors critically revised the manuscript, read, and approved the final version.

The authors apologize for this error and state that this does not change the scientific conclusions of the article in any way. The original article has been updated.

